# Individual Differences in Sequential Movement Coordination in Hip-Hop Dance: Capturing Joint Articulation in Practicing the Wave

**DOI:** 10.3389/fpsyg.2021.731901

**Published:** 2021-10-27

**Authors:** Derrick D. Brown, Guido Wijffels, Ruud G. J. Meulenbroek

**Affiliations:** ^1^Donders Institute for Brain, Cognition and Behaviour—Donders Centre for Cognition Nijmegen, Radboud University Nijmegen, Nijmegen, Netherlands; ^2^Donders Institute for Brain, Cognition and Behaviour, Radboud University Nijmegen, Nijmegen, Netherlands

**Keywords:** attentional foci, dance and movement, hip-hop, individual differences, motor processes (2330), motor control

## Abstract

The current study highlights individual differences in the joint articulation strategies used by novices practicing a hip-hop dance movement, the wave. Twelve young adults, all naive regarding hip-hop dance performance, practized the wave in 120 trials separated into four blocks with the order of internal or external attentional focus counterbalanced across subjects. Various kinematic analyses were analyzed to capture performance success while exploiting the observed individual differences in order to establish the reliability of the proposed performance indicators. An external focus of attention marginally facilitated the smooth transfer of a wave motion across neighboring limb segments as characterized by a constant propagation speed combined with large wave amplitudes. Systematic correlations between the success indicators were found, exemplifying the various degrees of joint articulation that novices prove capable of during an initial practicing session to try and perform a novel complex motor task.

## Introduction

Kinematic analyses of dance movements have traditionally focused on movements of the lower limbs, such as turning, jumping, and elevation of the legs, often to highlight injury profiles and improve rehabilitation and performance (Miura et al., 2011; Chia-Wei et al., 2014). Movements of the upper extremities in dance have received less attention in research even though they are seen as a hallmark of dance performance across genres. In non-dance circles, a perhaps salient example of the upper body would be the flapping wing-like arm sequences seen images of the Dying Swan (Kawano and Kuno-Mizumura, 2019), or the intricate arm movements in Flamenco (Forczek et al., 2017). Recently, there has been a growing interest in analyzing upper limb movement kinematics in hip-hop dance (Sato et al., 2010, 2015; Tsiouti et al., 2016). The studies mentioned here reflect a possibility that upper-body kinematics reveal more about the aesthetic and qualitative demands of dance than a dance-injury construct often seen in lower body kinematics. The aim of the current study is to extend the research of Sato et al. (2015) to naïve participants practicing a hip-hop dance wave motion through the fully and horizontally stretched arms with the additional goal to explore behavioral dimensions which may serve a functional role in feedback and or knowledge-of-results based dance training. The latter target, however, requires further empirical studies.

**FIGURE 1:**
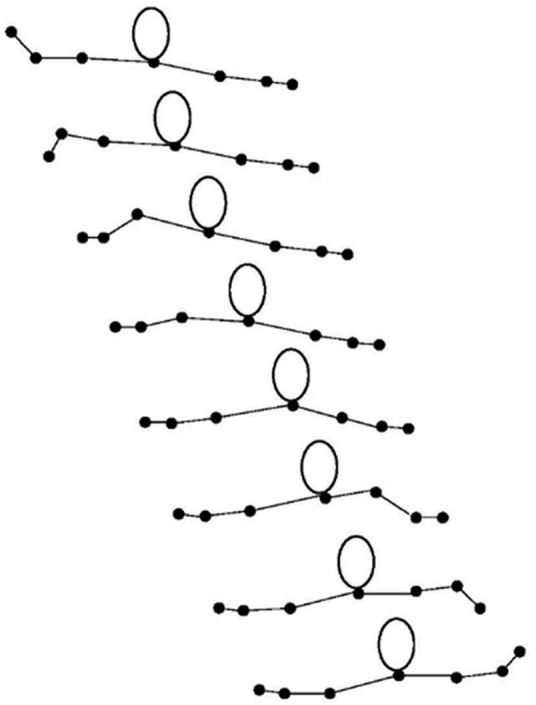
The left-to-right hip-hop wave represented by eight line drawings taken (from to bottom) equidistantly in time from a video rendering. In addition, an IRED was attached to a headset atop of the head as a reference of location.

A movement that is novel to many and consists of sub-movements of the arms chained together is the “wave.” The wave is a move in hip-hop dance (Figure 1) in which the dancer gives the impression that a wave rolls through the upper body (Sato et al., 2015). Whilst there are many types of “waves” in hip-hop, here we operationalize a wave that is moving smoothly in sequence with no rhythmic or physical accents (Sato et al., 2010). The wave moves in succession or with systematic degrees of overlap, across the frontal plane along the horizontal axis of the body. The wave investigated here consists of eight sub-movements when considering the arm segments and joints involved. Thus, from the hand (1), through the wrist (2), then elbow (3); through the first shoulder (4) then the second shoulder (5); through the second elbow (6), then the second wrist (7) and ending at the second hand (8). When a specific part of the chain is in action, other parts should ideally remain stationary.

Consequently, a wave necessitates task-orientated multi-joint synergy with direct or indirect control over a joint’s (or segment’s) with maximum amplitude and the speed of the rotating joint segment. In contrast, the other joints of the chain produce minimal movement or even remain motionless. Keeping parts of the kinematic chain stationary is complicated for two reasons. Firstly, because our limbs move via mono- and polyarticular muscles covering single or multiple joints. When polyarticular muscles contract, the effector system facilitates synergies across multiple joints (Schillings et al., 1998; Goble et al., 2007). In the wave, however, it is expected that the dancer moves the joint segments sequentially. Secondly, interactive torques passively occur in a chain when single joints move. These torques create an additional constraint when attempting to minimize movement at distal segments in the chain. Indeed, if one extends the upper arm with force, interaction moments between the shoulder, elbow and wrist will elicit the hand to extend as well (Galloway and Koshland, 2002). Keeping the hand motionless while moving the arm thus requires substantial neutralization or compensation of the inter-joint interactions due to muscular interference (Meulenbroek et al., 1998; Dounskaia et al., 2005; Bosga et al., 2010). With respect to inter-joint coordination, a successfully performed wave in this study is one in which identifiable offsets of joint movement initiation, lead to a smooth transfer of wave motion across neighboring limb segments. We intend to assess the quality of variations in various *amplitude* and *duration parameters* of the wave in the kinematic chain while varying the instructional constraints.

Visual and verbal instructions are informational constraints used to transfer task knowledge (Spray and Newell, 1986). Key in this theoretical model is the concept of knowledge of results. Thus, within the results of a time-series analysis, systematic shifts in performance from trial to trial are typically expected as the performer improves with practice (Newell, 1976). In the current study, knowledge of results is communicated in terms of movement instructions that refer to the arm segments rather than static targets. Thus, verbalizing a task with added visual demonstration offers constraints that shape the learner’s understanding of task goals. In dance education, the opposite finding appears salient; learning dance movement initially from observation is more beneficial than verbal instruction (Gray and Skrinar, 1984; Bläsing et al., 2018). Whiting and Brinker (1982) put forward the idea that learners utilize information that facilitates movement imagery of the task (Whiting, 1988) and the potential focusing on the successful movement to be achieved. Consequently, when considering the effects of internal and external foci of attention on motor learning, skill level is an important consideration. Several studies in physiotherapy have tested two kinds of attentional foci to facilitate acquisition of new motor skills. For example, Emanuel et al. (2008) investigated whether the effect of attention focus varies among children and adults. Thirty-four children and 32 adults were randomly assigned to internal or external focus-of-attention practice groups in a dart throwing task. Their results indicated that focus of attention varies between children and adults in accuracy with external focus more effective than internal focus in adults and the recommendation that physiotherapist should instruct adult clients to focus their attention externally to facilitate motor learning. Rather than a therapeutic approach we investigate foci of attention from a learning perspective. An overarching observation is that when a novice focuses on internal proximities, e.g., limb orientation, or technical execution when learning a new task, the performance results are suboptimal, and automatic execution impeded. Conversely, directing attention externally, away from the body, to the distal effects the movements yield in the environment, appears to boost performance results in skill acquisition (for a review see Wulf, 2013). By and large the research would suggest that there are advantages to adopting an external vs. internal focus produced via informational constraints. However, it is worth noting that many of the studies in the review mentioned earlier, utilize movement paradigms that require the use of equipment for facilitating the task at hand. Thus, equipment-assisted tasks such as batting, dribbling, and juggling, all enable or in some cases initiate movement. Research in classical ballet has provided mixed results with no discernible (Denardi and Corrêa, 2013; Guss-West and Wulf, 2016) or negligible effect on performance when using an external focus (Chua et al., 2018). Secondly, many sports-type movements adopted in experimental protocols, involve familiar, action-based hitting, throwing or balancing, i.e., gross motor coordination movements for which participants presumably have *a priori*, procedural knowledge. Consequently, it may be difficult to generalize the effects of instructional constraints from sport-based movement modalities when characterizing genre specific dance movement (Peh et al., 2011). In a lab setting, whilst the parameters of many experimental tasks are often categorized as novel, the functional movements often under investigation are decidedly familiar (Bläsing, 2015). Thus, in the current study we query if an external attentional focus facilitates the performance of a wave pattern performed by a group of novices with no user knowledge in hop-hop dance.

The quality of the wave motion in hip hop can be assessed in multiple ways. A dancer can decide to make sub-movements large or small, isolated, or amalgamated, fast, or slow, a decision wholly dependent on subjective and aesthetic factors. However, expert hip-hop dance judges have rated waves with a constant propagation velocity as most favorable across hip-hop dancers of different levels (Sato et al., 2014). The judges’ performance scores were not correlated with the amplitude of movements or the low number of residual movements, i.e., movements in parts of the body expected to remain stationary. Hence, we wished to explore here whether these parameters would reveal the spatial quality of the wave produced by novices. In addition to constant propagation velocity, we also wished to explore the time it took the participants to complete the wave, or duration, associated with the amplitude of the wave movements in the kinematic chain also compared to the amplitude of residual (i.e., unwanted) movements. Our rationale here rests in the idea that a longer time to completion with unwanted movements thwart the attempt at multi-joint synergy (Morel et al., 2017). Moreover, we adopted an interindividual differences approach to capture the variations in novices’ motor learning strategies. By exploring the correlations between spatial and temporal performance measures on the one hand and propagation speed on the other, we wish to garner insight into which behavioral parameters might be exploited in future applications of our research in knowledge-of-result based dance training contexts (Blackwell and Newell, 1996; Ratcliffe and Newport, 2017; Pacheco and Newell, 2018).

In summary, in the present study, we explore spatiotemporal variables in the acquisition of a hip-hop dance wave motion pattern in novices due to instructional constraints. In light of insufficient evidence of the effects of instructional constraints on novel dance movements in novices, we hypothesized that an external focus of attention would have no significant effect across the trials on performance of the wave in our novice group of participants. We expected that participants with a more constant propagation speed would show a smoother transfer across neighboring joint segments regarding associations in our spatiotemporal parameters. Finally, we differentially explored the relationships between the relative spatial prominence of the wave motion in terms of vertical amplitudes of arm segments and duration of the wave across individuals to gain insight into potentially and didactically relevant instructional dimensions.

## Materials and Methods

### Participants

Twelve participants (5 males, 7 female) volunteered to take part in this experiment (mean (*SD*) 22 (*2*) years). Participants were students from the Radboud University, Nijmegen. All participants declared they were naïve to the hip-hop dance waving task.

Before the experiment commenced, participants provided informed written consent and filled out a PAR-Q (Thomas et al., 1992) and provided information on dance experience. None of the participants reported any physical problems that would impact their ability to participate. The research protocol was in agreement with the Helsinki Declaration and the study was approved by the ethics committee of the Faculty of Social Sciences of the Radboud University, Nijmegen.

#### Instruction Protocol

The instruction protocol was comprised of video viewing, written wave instruction, and written foci of attention definition instruction. Participants were shown an 11-sec excerpt of video in which only the segment of arm-wave tutorial is demonstrated for dance education purposes (Steffanina, 2013). The video sound was muted, thus only a visual instruction was provided. Participants viewed the video a maximum of 10 times in one sitting.

After viewing the video, participants were given a written description of a hip-hop wave and how their performance would be assessed. Successively, participants were requested to recite the meaning and their language comprehension of the written instruction.

The written instructions were:

(1) using a constant speed, i.e., no pauses during the sequence but one smooth movement from your left-hand to your right-hand (and vice versa).

(2) generate a full wave, meaning, the arm parts that should move, move as large as possible and parts that should remain still, move as little as possible.

Regarding attentional instructions, participants were then asked to read and demonstrate their comprehension of the following sentences:

Internal focus

*While performing the movement, imagine your arms moving up and down sequentially, starting with your first arm, the hand, then your elbow, your shoulder, then the shoulder, elbow, and hand on your other arm.*

External focus

*While performing the movement, imagine and follow a ripple in a rope that is being whipped once. This causes the rope to move up and down*.

#### Task and Design

Participants were seated comfortably on a cushioned stool which facilitated 90-degree flexion of the knee with feet resting comfortably on the floor. Height adjustments were made mechanically to the chair when necessary. Participants had an unobstructed visual field and there were no mirrored surfaces in the laboratory. They performed 120 trials separated into four blocks of 30 trials each. Before each block, participants were informed of the attentional focus expected for the block and asked to repeat their comprehension of that focus of attention, if necessary, the experimenter clarified their understanding. The order of attentional instruction was counterbalanced across participants using design. Thus, participants were divided in two different groups, one following the ABBA design and the second one the BAAB design. Between the four experimental blocks, participants were allowed 5 min of rest. In 5% of the trials missing data were due to IREDs having moved out-of-range were found. These data were interpolated with spline functions, thus reducing the missing data that were excluded from further analyses to less than 3%.

Each trial consisted of one wave starting either from the left-hand ending in the right-hand or in the opposite direction with the orientation of the focus of attention for each block according the ABBA or BAAB design. Therefore, attentional foci direction was alternated after each trial block (see also Figure 3). Participants started in the seated position with the hand relaxed with the palmar surface of the hand facing the cameras.

**FIGURE 2:**
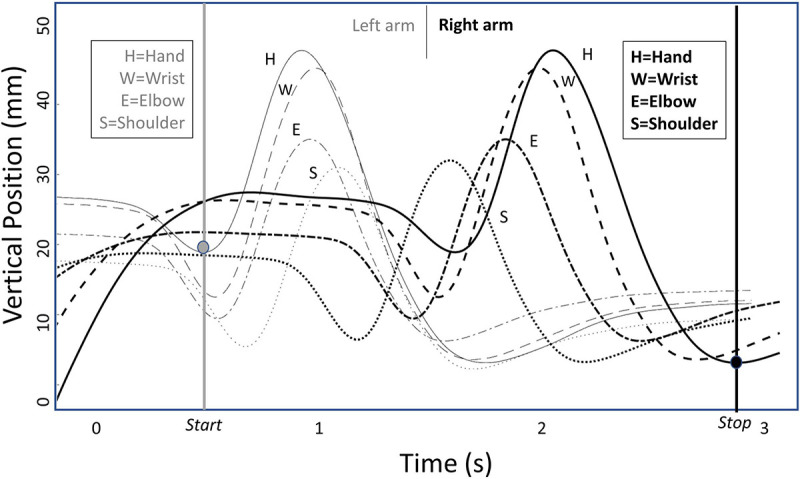
Prototype of pre-processed data of the wave selected in a 3 s position- time window. The *Y*-axis denotes the vertical position- time data equivalent to the height dimension. The graph depicts the complete wave in one direction starting with the left arm followed by the right arm.

Auditory signals indicated the preparation of the trials; three low pitch beeps followed by one higher pitch beep. Participants raised their arms to shoulder level within the first three beeps and were free to start their wave after the high beep. Following the directions of the experimenter participants could lower their arms back in the rest position after the experimenter judged the movement as completed. The experimenter provided no visual guides to serve as imitation throughout the trials. The rest between trials was maximally 5 s.

#### Apparatus and Data Collection

The movement sequences were measured by a 3D motion tracking system (OPTOTRAK 3020, Northern Digital Inc., Waterloo, Canada) at 100 Hz and a spatial accuracy of 0.2 mm in X, Y, and Z. We utilized nine infrared-emitting diodes (IREDs) placed on body segments relevant to the wave (Figure 1). In addition, an IRED was attached to a headset atop of the head as a reference location and was not a part of the data analysis. The infrared signals of the IREDs were captured by two cameras on the lateral sides of the participants angled from above. Data consisted of position coordinates in time and was thus deidentified.

**FIGURE 3:**
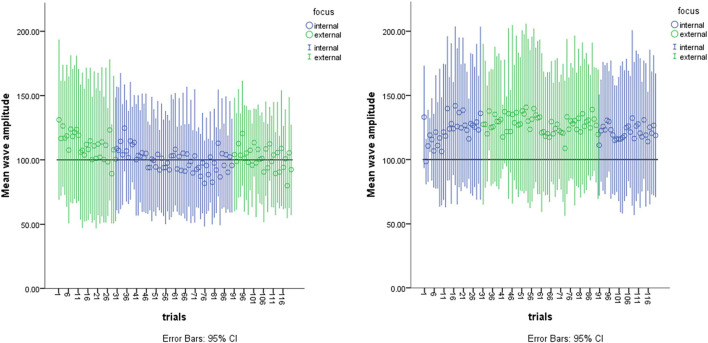
Sequential Effects on mean wave amplitudes of Instruction type and trials. The left-hand graph shows the data of the participants who performed the ABBA sequence where A = External Focus of Attention and B = Internal Focus of Attention. The right-hand graph shows the data of the participants who performed the BAAB sequence.

### Data Analysis

Movement data were analyzed with MATLAB^®^ (R2017a). A cubic spline interpolation was used to interpolate between unknown data points resultant from participants turning away IREDs from camera view during the wave. This was necessary for to reach maximum amplitudes in the wave it helps to rotate the elbow joint around the longitudinal (prosupination) axis in the arm. Data analysis was limited to only motions in height. Individual trials were plotted, and the start and end point of the wave were manually set.

The waveform was revealed in the raising and lowering of each IRED in a specific order as seen in Figure 2 below. Therefore, the successive peaks of the vertical IRED displacements allowed the segmentation of the wave. The individual peaks of each IRED during each trial were used to calculate target variables based on instruction and the formulated hypotheses. The amplitude of the segments was calculated by the *prominence* of its peak height, which is the most visible upward peak within the analyzed window. For each segment of the kinematic chain used to perform the wave an amplitude (in mm) was calculated. Of the segments intended to not show movement, the amplitudes were also calculated and labeled as amplitudes of the residual movements. Of each sub movement the duration was denoted in seconds. The sum of these durations yielded the wave duration (in seconds) and the mean duration of the sub movements inversely indexed the average propagation speed. More importantly, we calculated the coefficient of variation (COV) of the propagation speed in line with Sato et al. (2015), i.e., the standard deviation of the sub movement durations within a trial divided over the mean sub movement duration, multiplied by a 100 to arrive at a COV expressed as a percentage. A constant propagation speed was thus reflected by a low COV percentage.

#### Statistical Analysis

Descriptive statistics for the kinematic variables were conducted using SPSS statistical package (version 25; SPSS Inc., Chicago, IL). Correlational analysis was performed with Pearson’s *r*. Finally, to test the validity of our kinematic variables *post hoc*, a Spearman’s rank order correlation coefficient analysis was conducted to analyze unsuccessful-successful performance. Effects of Focus of Attention was evaluated with a one-way ANOVA design in which the effect strengths was assessed by calculating eta squared values. Level of statistical significance was set at *p* < 0.05.

## Results

### Attentional Focus

We predicted that an external focus would have no impact on the execution of a dance wave pattern in novice individuals. The one-way ANOVA showed no difference in the wave pattern performance as a function of attentional focus [*F*(1, 11) = 3.25, *p* = 0.10, eta = 0.227]. Only a marginally higher amplitude was revealed in the external focus (*M* = 115, *SD* = 41.75) vs. the internal focus (*M* = 109, *SD = 37.99*). Figure 3 shows the sequential effects of instructional types across the trial blocks and trials.

**FIGURE 4:**
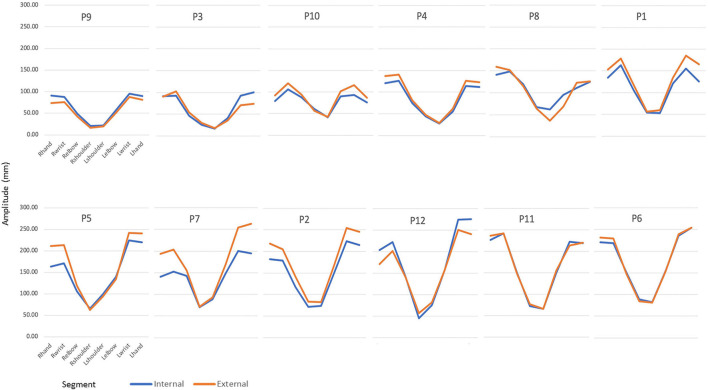
Sequential joint articulation and differentiation expressed in terms of internal (blue) and external (orange) foci of attention. The hand amplitudes in all participants were larger than the shoulder amplitudes. Differences can be seen in a small and modest wave pattern (participant 9) vs. a large more pronounced wave pattern (participant 6).

We ranked the participants on the wave-amplitude dimension to explore the individual coordination strategies taken to complete the task. Figure 4 reveals the amplitude articulation during the wave task across the arm segments for each participant, ranked from having generated small amplitudes (participant 9; top-left panel) to having generated large amplitudes (participant 6; bottom-right panel).

### Correlations

Table 1 shows the overall correlations between the dependent measures. The mean wave amplitude correlated positively with the difference between the wrist and shoulder amplitudes (Rxy = 0.887) but negatively with the coefficient reflecting the constancy of the propagation speed (Rxy = −0.770). The latter correlation reflected those participants who succeeded in producing large wave movements failed to generate a smooth wave sequence exhibiting a constant progression speed. The wave quality is also shown by the negative correlation between the residual amplitude and the wave over the residual amplitude ratio (Rxy = −0.851). The residual amplitude ratio expressed in the movement segments intended to move the least, is a signal to noise ratio since the wave amplitude can be regarded as the target, i.e., parameter to be expressed, while keeping amplitudes in the other (residual) segments to a minimum. The statistically significant negative correlation between the wave over the residual amplitude and the wave duration (Rxy = −0.579) showed that successful participants also managed to generate the wave at a high speed. Participants generating large amplitudes while managing to keep residual amplitudes small, hence generated salient wave patterns of shorter durations. Thus, participants who were successful, not only produced large waves but managed to do so at high speed. As regards performance speed, the statistically significant positive correlation between wave duration and the duration of the should-to-shoulder interval (Rxy = 0.883) demonstrated a chunking coordination strategy consisting of first generating the wave in one arm followed by a short break during which a head movement might have occurred in a relatively long shoulder-to-shoulder interval, after which the wave of the second arm was generated. The latter correlation thus reflects the participants were novices and failed to maintain a constant progression speed particularly when the wave needed to be continued at the chosen speed from one arm to the other.

**TABLE 1:** Pearson’s correlations of the chosen kinematic variables (see text).

	Wave amplitude	Wrist minus shoulder amplitude	Residual amplitude	Wave/residual amplitude ratio	Shoulder to shoulder interval	Duration	COV propagation speed
Wave amplitude	1	0.887**	0.133	–0.046	0.299	0.179	−0.770**
Wrist minus shoulder amplitude		1	0.093	0.094	0.006	0.017	−0.773**
Residual amplitude			1	−0.851**	0.336	0.330	0.220
Wave/residual amplitude ratio				1	–0.569	−0.579*	–0.415
Shoulder to shoulder interval					1	0.883**	0.169
Duration						1	0.250
COV Propagation speed							1

**Correlation is significant at the 0.01 level (2-tailed).

*Correlation is significant at the 0.05 level (2-tailed).

### Exploratory Spatial Correlations

**FIGURE 5:**
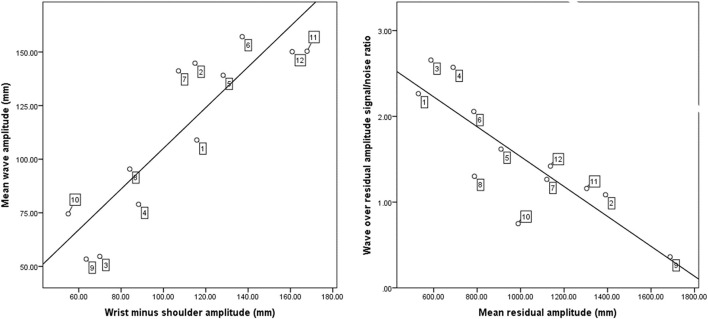
Scatter plot on the left reveals the relationship between the wave amplitude and the wrist minus the shoulder amplitude. The scatter plot on the right reveals the relationship between the residual amplitude and the signal to noise ratio of wave amplitude over the residual amplitude. The numbers anchored to the data points along the regression line denote the individual participants designation (e.g., 10 = participant 10). The position of participant designation denotes from top-left to bottom-right in the graph the ranking of more successful to least successful performers.

Figure 5 shows in a two-panel graph the results of our exploration of the between-subject variations in some additional spatial parameters relevant to our participants’ performance success. The left panel shows the relationship between the wave amplitude and the wrist minus the shoulder amplitude, *r* = 0.887, *P* = 0.000. The right panel shows the relationship between wave over residual amplitude ratio on the *Y*-axis and the mean residual amplitude on the *X*-axis, *r* = −0.851, *P* = 0.000. If we presume that a pronounced amplitude differentiation across their proximal and distal arm segments relates to well-articulated waves with successful inter-joint coordination (Goble et al., 2007), then participants in the top-right quadrant in the left panel of Figure 5 performed a well-articulated wave, (i.e., participants 6, 11, and 12) whilst those in the bottom-left quadrant in the left panel were least successful in the segmental differentiation in the wave pattern (i.e., participants 3, 9, and 10). In the right panel of Figure 5 participants in the bottom-right quadrant produced a smaller signal-to-noise ratio with a larger residual amplitude (i.e., participants 2, 9, and 11) whilst participants in the upper-left quadrant were more successful, i.e., they managed to produce larger signal-to-noise amplitude ratios while keeping the amplitudes of the residual (non-wanted) movements small (i.e., participants 1, 3, and 4).

### Exploratory Temporal Correlations

**FIGURE 6:**
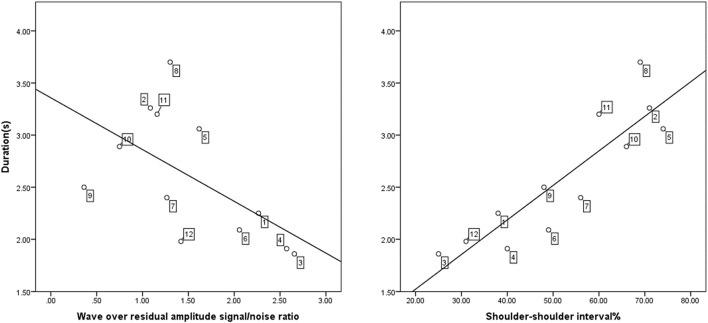
The scatter plot on the left reveals the relationship between the signal to noise ratio of wave amplitude over the residual amplitude and the duration in seconds. The scatter plot on the right, reveals the relationship between the duration in seconds and the shoulder-to-shoulder interval. The numbers anchored to the data points along the regression line denote the individual participants designation (e.g., 10 = participant 10). The position of participant designation denotes the ranking of more successful (e.g., 6, 12) to least successful (e.g., 8, 11) performers.

Figure 6 reveals in a 2-panel graph the relationship of three temporal parameters. The left panel shows the relationship between wave duration and the amplitude signal-to-noise ratio, *r* = −0.579, *P* = 0.049. The right panel shows the relationship between wave duration and shoulder-to-shoulder relative time, *r* = 0.883, *P* = 0.000. Thus, as seen in the left panel of Figure 6 participants who generated large wave over residual amplitude ratios needed less time and were thus more successful in the wave pattern (i.e., participants, 1, 3, 4, and 6). Regarding the shoulder-to-shoulder interval and time to completion (Figure 5 right panel), participants with a low percentage between the shoulder segments took less time to complete the wave (i.e., participants 3, 4, and 12) whilst those with a higher percentage of time between the shoulder segments took longer to complete this segment of the wave (i.e., participants 2, 8, and 11).

## Discussion

The purpose of this study was to investigate spatiotemporal variables in novices performing during a first practicing session an unfamiliar dance-wave motion pattern under two different instructions. To this end we conducted kinematic analyses of self-generated, upper body hip-hop wave patterns in individuals with no prior dance experience. Regarding attentional focus we hypothesized that an external attentional focus would have no significant effect across the trials on performance of the wave. With an alpha level of *p* = 0.10 the results revealed no statistical significance but a weak trend toward the external attentional focus yielding marginally better performance. In terms of individual attentional strategies, the participants who started with the external focus (left graph Figure 3) tended to *reduce* their wave amplitudes as the trial series progressed whereas the participants who started with the internal focus (right graph Figure 3) tended to *increase* their wave amplitude as the trial series proceeded. Interestingly, in the first trial blocks the participants who initially employed an internal focus managed to generate larger, albeit marginal, wave amplitudes (right-hand graph in Figure 3) than the participants who started initially with an external focus (left-hand graph in Figure 3). This group difference is at odds with the expectation of external attentional focus facilitating performance (Wulf, 2013). Furthermore, we also ranked the participants on the wave-amplitude dimension to explore the individual strategies in sequential joint articulation and differentiation taken to complete the task. As seen in Figure 4, smaller amplitudes were performed by participant 9 whilst larger amplitudes were performed by participant 6. All participants showed varying degrees of joint articulation resulting from the attentional foci. This would imply that interindividual differences in a novice cohort should not be categorized as noise, rather as a behavioral strategy used particularly in earlier stages of learning. Moreover, we specifically studied a novel task, dance, in a non-dance population. The rationale here was to corroborate the recommendation by Peh et al. (2011) to investigate attentional focus on participants with no *a priori* intrinsic dynamics and preferred movement coordination patterns. We confirmed prior to data collection that our participants were indeed novices to dance and this specific hip hop movement pattern. Thus, in the current study we can substantiate the benefit albeit marginal of an external instructional foci on novices performing a novel movement task.

With respect to spatiotemporal relationships, we sought to extend research done by Sato et al. (2015). To that end we empirically tested the relationship between a constant propagation velocity and the size of the wave prominence; a larger wave amplitude equating to a more successful wave performance. In line with Sato et al. (2015), we found a significant negative correlation between the wave amplitude and the coefficient of variation (COV) of the propagation speed. The novices who managed to maintain a low COV of propagation speed also produced larger wave amplitudes as shown in Figure 5. Thus, successful participants managed to satisfice both the spatial and temporal task constraints.

In our exploratory analyses of spatial parameters we noted between-participant variations across limb segments and size of amplitude. This finding highlights the compensatory strategies present in coordinating interaction moments across limb segments in an effort to reduce superfluous movement during the task (Galloway and Koshland, 2002). As shown in the right panel of Figure 5, participants who generated small residual segment amplitudes (i.e., noise) generated large wave amplitude over residual amplitude ratios. Qualitatively thus, a successful wave was generated by participants who kept the residual movements small. As seen in the left panel of Figure 5 those participants who showed a pronounced amplitude differentiation across their proximal and distal arm segments, also managed in generating salient wave amplitudes, and thus successfully articulated the wave pattern. In summary, these relationships extend the research of Sato et al. (2015) and highlight the potential that explicit, individual feedback can provide the learner. The multiple individual strategies adopted during cognitive and associative phases of learning may increase behavioral flexibility (Stanton, 2011; Ranganathan et al., 2020) and facilitate the overall result of movement production. Future studies could further elucidate that intricacies of interjoint articulations with respect to the strategies adopted by novices when learning a complex arm movement. These findings emphasize the interindividual differences in aesthetic quality whilst learning, which are important in a highly individualized and variable setting as hip-hop dance training.

Regarding the correlations between temporal performance variables, we assessed the relationship between the signal to noise ratio of wave amplitude over the residual amplitude, and shoulder to shoulder intervals when correlated with time to completion of the wave. Here, we observed that participants who generated large wave over residual amplitude ratios needed less time to generate the wave pattern. Thus, as illustrated in Figure 6, more successful performers therefore took relatively less time to complete the wave. These differential movement strategies are in line with minimization principles research which suggest that a longer duration to complete a movement task requires more effort (Morel et al., 2017), which in turn appears reliant on skill level (Beilock et al., 2008), particularly in spatiotemporal task such as dance (Sgouramani and Vatakis, 2014). Consequently, skill, or lack thereof as revealed in our novice cohort, is a determining attribute when applying foci of attention as seen in research with ballet dancers (Denardi and Corrêa, 2013; Guss-West and Wulf, 2016) a dance genre different in training and execution to hip-hop dance in this study. The cognitive load present during skill acquisition is critical when instructing novices and beginners. Instructors can provide students with critical feedback on the relative timing of the movement segments to mitigate the onset of fatigue in the early stages of learning and refining this movement (Tsiouti et al., 2016). Regarding the shoulder-to-shoulder interval we observed that participants who completed the wave segmentation between the shoulders quickest in the overall time duration were more successful in the wave task. This finding appears in line with the observation of differentiation in joint coordination and dynamics, particularly in the horizontal plane, most manifest at the shoulder-to-shoulder segment of the movement task (Galloway and Koshland, 2002). Thus, irrespective of the timing duration albeit less or more time, instructional feedback provided on the temporal smoothness of the movements at the shoulder segment can assist novices in a successful movement performance.

Finally, the observation of statistically significant correlations which our explorative analyses of spatial and temporal kinematic parameters yielded, support the validity of the applied measures on a unsuccessful-successful scale reflecting the degree of performance success as described above. The rankings of the participants on this scale as portrayed in Figures 4–6 revealed in a *post hoc* spearman-Rho rank-order correlation analyses to yield only a single positive correlation, namely that between the rank orders displayed in Figure 4 and the left-hand graph of Figure 5 (Spearman Rho = 0.65, *n* = 12, *p* = 0.065). All other rank-order correlations (*N* = 9) proved statistically non-significant, underscoring that learning a hip-hop dance move as studied here is a highly individualized process, i.e., success of a pupil (relative to the success of peers) on one performance dimension does not guarantee (equal) success on another dimension.

## Conclusion

In this study we scrutinized a sequential joint-articulation task, i.e., the hip-hop wave pattern in a non-dance population, performed for the first time under different instructions as regards attentional foci. We found that in half the participants who initially focused attention internally, performance was marginally facilitated. Further, we corroborated previous research on the relationship between constant propagation speed and larger wave amplitudes as indices of successful performance. Finally, we highlighted relationships between timing aspects and the intricacies of inter-joint coordination as potentially important in dance education when teaching a hip-hop arm wave exercise because we related interindividual performance differences to greater and less well articulated dance performance.

## Data Availability Statement

The raw data supporting the conclusions of this article will be made available by the authors, without undue reservation.

## Ethics Statement

The studies involving human participants were reviewed and approved by the Social Sciences Faculty of the Radboud University, Nijmegen, Netherlands (approval code: ECSW-2018-010R1). The patients/participants provided their written informed consent to participate in this study.

## Author Contributions

DB, GW, and RM: conceptualization, writing—original draft, writing—review, and editing, formal analysis. DB and GW: investigation and methodology. DB and RM: visualization. All authors contributed to the article and approved the submitted version.

## Conflict of Interest

The authors declare that the research was conducted in the absence of any commercial or financial relationships that could be construed as a potential conflict of interest.

## Publisher’s Note

All claims expressed in this article are solely those of the authors and do not necessarily represent those of their affiliated organizations, or those of the publisher, the editors and the reviewers. Any product that may be evaluated in this article, or claim that may be made by its manufacturer, is not guaranteed or endorsed by the publisher.
