# Corrigendum: Individual Differences in Sequential Movement Coordination in Hip-Hop Dance: Capturing Joint Articulation in Practicing the Wave

**DOI:** 10.3389/fpsyg.2021.830112

**Published:** 2022-01-11

**Authors:** Derrick D. Brown, Guido Wijffels, Ruud G. J. Meulenbroek

**Affiliations:** ^1^Donders Institute for Brain, Cognition and Behaviour—Donders Centre for Cognition Nijmegen, Radboud University Nijmegen, Nijmegen, Netherlands; ^2^Donders Institute for Brain, Cognition and Behaviour, Radboud University Nijmegen, Nijmegen, Netherlands

**Keywords:** attentional foci, dance and movement, hip-hop, individual differences, motor processes (2330), motor control

In the original article, there was an error. In the description of the results displayed in Figure 3 in the first paragraph of the discussion section, the references to the experimental conditions with respect to internal and external focus of attention were erroneously switched.

A correction has been made to *Discussion, Paragraph 1*. The corrected paragraph is shown below.

The purpose of this study was to investigate spatiotemporal variables in novices performing during a first practicing session an unfamiliar dance-wave motion pattern under two different instructions. To this end we conducted kinematic analyses of self-generated, upper body hip-hop wave patterns in individuals with no prior dance experience. Regarding attentional focus we hypothesized that an external attentional focus would have no significant effect across the trials on performance of the wave. With an alpha level of *p* = 0.10 the results revealed no statistical significance but a weak trend toward the external attentional focus yielding marginally better performance. In terms of individual attentional strategies, the participants who started with the external focus (left graph Figure 3) tended to *reduce* their wave amplitudes as the trial series progressed whereas the participants who started with the internal focus (right graph Figure 3) tended to *increase* their wave amplitude as the trial series proceeded. Interestingly, in the first trial blocks the participants who initially employed an internal focus managed to generate larger, albeit marginal, wave amplitudes (right-hand graph in Figure 3) than the participants who started initially with an external focus (left-hand graph in Figure 3). This group difference is at odds with the expectation of external attentional focus facilitating performance (Wulf, 2013). Furthermore, we also ranked the participants on the wave-amplitude dimension to explore the individual strategies in sequential joint articulation and differentiation taken to complete the task. As seen in Figure 4, smaller amplitudes were performed by participant 9 whilst larger amplitudes were performed by participant 6. All participants showed varying degrees of joint articulation resulting from the attentional foci. This would imply that interindividual differences in a novice cohort should not be categorized as noise, rather as a behavioral strategy used particularly in earlier stages of learning. Moreover, we specifically studied a novel task, dance, in a non-dance population. The rationale here was to corroborate the recommendation by Peh et al. (2011) to investigate attentional focus on participants with no *a priori* intrinsic dynamics and preferred movement coordination patterns. We confirmed prior to data collection that our participants were indeed novices to dance and this specific hip hop movement pattern. Thus, in the current study we can substantiate the benefit albeit marginal of an external instructional foci on novices performing a novel movement task.

Corrections have also been made to *Conclusion, Paragraph 1*.

In this study we scrutinized a sequential joint-articulation task, i.e., the hip-hop wave pattern in a non-dance population, performed for the first time under different instructions as regards attentional foci. We found that in half the participants who initially focused attention internally, performance was marginally facilitated. Further, we corroborated previous research on the relationship between constant propagation speed and larger wave amplitudes as indices of successful performance. Finally, we highlighted relationships between timing aspects and the intricacies of inter-joint coordination as potentially important in dance education when teaching a hip-hop arm wave exercise because we related interindividual performance differences to greater and less well articulated dance performance.

The authors apologize for these errors and state that they do not change the scientific conclusions of the article in any way. The original article has been updated.

## Publisher's Note

All claims expressed in this article are solely those of the authors and do not necessarily represent those of their affiliated organizations, or those of the publisher, the editors and the reviewers. Any product that may be evaluated in this article, or claim that may be made by its manufacturer, is not guaranteed or endorsed by the publisher.
